# Ramadan Fasting and Maternal and Fetal Outcomes in Pregnant Women with Diabetes Mellitus: Literature Review

**DOI:** 10.3389/fendo.2022.900153

**Published:** 2022-06-24

**Authors:** Shejil Kumar, Terrence Diamond

**Affiliations:** Endocrinology Department, St George Public Hospital, Sydney, NSW, Australia

**Keywords:** Ramadan, fasting, diabetes, gestational diabetes, type 2 diabetes, maternal outcomes, fetal outcomes

## Abstract

There is an emerging Muslim and diabetic population in the United States and other Western countries and majority of pregnant women and patients with diabetes mellitus choose to fast during Ramadan. Fasting during Ramadan in pregnant women with diabetes may represent a ‘perfect storm’ of metabolic disturbances including hyperglycemia, hypoglycemia and ketosis. Recent continuous and flash glucose monitoring data suggests increased glycemic variability (fasting hypo- and post-Iftar hyperglycemia) in non-pregnant patients with diabetes during Ramadan. Only five small-scale studies, predominantly focused on women with gestational diabetes mellitus in Muslim-majority nations have explored maternal glycemic outcomes during Ramadan which is associated with lower mean blood glucose levels and higher frequency of fasting hypoglycemia. Data is limited however on important clinical outcomes such as symptomatic and serious hypoglycemia requiring hospitalization. Results have been conflicting regarding maternal Ramadan fasting and association with fetal outcomes in women without diabetes. Only one recently published study reported on perinatal outcomes in pregnant women with gestational diabetes which found no effect of Ramadan exposure on mean birthweight or macrosomia frequency but lower neonatal hypoglycemia prevalence, however a significant limitation was lack of documentation of maternal fasting status. At this stage, due to paucity of data, the current medical recommendation is against Ramadan fasting for pregnant Muslim women with diabetes. Large-scale population-based studies are warranted regarding maternal and fetal outcomes in pregnant fasting women with diabetes and such studies should characterize maternal fasting status and have meaningful and consistent clinical outcomes. High-quality data derived from these studies can assist clinicians in providing more evidence-based advice to safely navigate both mother and fetus through a potentially challenging pregnancy.

## Introduction

There is little evidence to guide diabetes management in Muslim pregnant women fasting during Ramadan, particularly in the Western World. There has been a literature surge in the past 5 years with majority of data from Muslim-majority countries such as the United Arab Emirates (UAE). Utilizing the USA as an example, as of 2017, according to estimates from population surveys, there were 3.45 million Muslims in the United States (1.1% of the population), an increase from 2.75 million in 2011 with Islam projected to be the fastest growing religion (1). In 2018, diabetes affected 10.5% of the population (34.2 million Americans) with an incidence of 1.5 million Americans per year (2). In 2016, the estimated national prevalence of gestational diabetes was 6.0%, an increase from 3.7% in 2000 (3). Individuals from South-East Asian and Middle Eastern descent have a higher diabetes prevalence (4). Further, Ramadan intersects with majority of all pregnancies. Hence, this is a significant emerging issue physicians will face in Western countries and there is an urgent need for more high-quality data and better physician understanding and cultural sensitivity to facilitate superior, evidence-based management for these women.

## Methods

This narrative review critically evaluates existing data regarding effects of Ramadan fasting on a) non-pregnant patients with diabetes, and b) pregnant women with diabetes, as well as fetal outcomes associated with maternal Ramadan fasting in mothers with and without diabetes.

We conducted extensive literature review through PubMed/MEDLINE database using search items: “Ramadan AND pregnancy”, “Ramadan AND diabetes”, “Ramadan AND gestational diabetes”, “Ramadan AND weight”, “Ramadan AND dietary intake”, “Ramadan AND nutrition”, “Ramadan AND maternal outcomes”, “Ramadan AND fetal outcomes”, “Ramadan AND hypoglycemia”, “Ramadan AND hyperglycemia”, “Ramadan AND ketosis”, “Ramadan AND ketoacidosis”, “Ramadan AND patient beliefs” and “Ramadan AND patient expectations”. Full texts of relevant articles were screened for inclusion. Further sources were derived from lists of similar articles and article reference lists.

## Background

### Ramadan – Religious Significance and Exemptions

Fasting during the holy month of Ramadan is one of the five pillars of the Islamic faith and is considered the month when the *Qur’an* was first revealed to the prophet Muhammad. Eating, drinking, smoking and sexual activity are prohibited between the sunrise and sunset hours which can range between 12 to 20 hours depending on geographic location and season (5, 6). The typical day during Ramadan for Muslims includes the pre-sunrise meal “Suhur” (5, 6), followed by a prolonged fast culminating in breaking the fast with the post-sunset meal “Iftar” (5, 6), traditionally celebrated with a large gathering with high intake of carbohydrate- and fat-rich food. Assessment of altered dietary patterns during Ramadan fasting is surprisingly limited (7–9). Studies have found conflicting results regarding changes in daily caloric, carbohydrate and fat intake during Ramadan, however majority of daily caloric consumption (up to 75-80%) has been shown to be consumed at Iftar in a study of 276 obese women with type 2 diabetes mellitus (T2DM) (7).

All healthy adult Muslims are obliged to fast during Ramadan. Religious exemptions from fasting include children, frail elderly, acute or chronic illness (e.g. diabetes), pregnant, lactating or menstruating women (10). However, small-scale surveys from Muslim-majority and Western countries indicate at least 80% of healthy pregnant women choose to fast (>50% for the entire month) despite exemptions, potential negative outcomes and health professional advice (11–14), while data on fasting frequency in women with GDM is conflicting (15, 16). The most common reasons for fasting include the spiritual desire to fast, sharing the connection with family and community, lack of recognition of potential dangers, lack of understanding of religious exemption from fasting, inconvenience of ‘making up the fast’ and potential guilt with not fasting (11–14). Regarding non-pregnant Muslims with diabetes, four large multinational landmark studies over the past 20 years indicate >80% of patients with T2DM intend to fast out of whom >50% complete the entire fast, compared to type 1 diabetes mellitus (T1DM), whereby 40-80% intend to fast and 25-50% fast the whole month of Ramadan (17–21).

### Ramadan – Perceived Benefits

Ramadan is also a time of prayer, reflection and community. Muslims may also derive physical benefits from fasting, such as weight loss. Two recent meta-analyses demonstrated significant modest weight loss (1.34kg, 95% CI 0.35-2.57kg; 1.24kg, 95% CI 0.88-1.60kg) pre-Ramadan to post-Ramadan although majority was regained within a few weeks and thus the transient weight loss is unlikely to have any meaningful clinical benefit (22, 23). One meta-analysis reported a reduction in fat percentage exclusively in overweight/obese people (1.46%, p = 0.010) which again returned to pre-Ramadan proportions within a few weeks (22). Another meta-analysis of 3,134 healthy participants found that glucose, insulin resistance, insulin, leptin and adiponectin levels were modestly reduced with Ramadan fasting, however studies demonstrated considerable heterogeneity and lacked controlling for potential confounders such as caloric intake, meal composition and physical activity (24).

Several small-scale population-based surveys on pregnant women and patients with diabetes reveal Muslims derive various social and spiritual benefits including increased self-control, compassion for those less fortunate, connection to religion, family and friends, and sense of spirituality and community (13, 25–28). There is also evidence Ramadan fasting is associated with improved parameters of mental, psychological and social wellbeing in individuals with and without diabetes, such as depression, anxiety, and diabetes-related distress (29–32).

### Ramadan and Diabetes – Cross-Cultural and Knowledge Gaps

Muslim pregnant women and patients with diabetes often withhold intention to fast from doctors and prefer discussion with family or community religious leaders for reasons including fear of disrespect or being told not to fast, lack of perceived clinician understanding of Ramadan, lack of recognition of right for fasting exemption and not feeling the need to seek professional advice (13, 15, 25, 27, 33). Several qualitative surveys of Muslims with diabetes and pregnant women from both Western and Muslim-majority nations have explored patient preferences regarding healthcare delivery surrounding Ramadan fasting (25–27, 34–36). These studies indicate a significant cross-cultural gap between non-Muslim physicians and their Muslim patients. Potential challenges include lack of physician knowledge and respect for Islamic religious and cultural beliefs, language/communication barriers, and patient lack of understanding of potential risks of fasting. A recently published cross-sectional survey explored physician perceptions (n = 260) of current knowledge gaps, barriers to research and potential future directions regarding Ramadan fasting and diabetes (37). Majority were senior diabetologists/endocrinologists (65.7%) working in tertiary centers (65.2%) in Muslim-majority countries, predominantly Middle East (49.6%) and Africa (29.2%). Ramadan fasting in pregnant women with diabetes was identified as the area needing most research (61.5%). Majority felt research methodology should focus on large epidemiological studies (49.5%) and double-blind placebo-controlled trials (48.6%) although we suspect the latter would be difficult to conduct in this setting.

### Physiology of Fasting in Healthy, Diabetic and Pregnant Individuals

**Figure 1:**
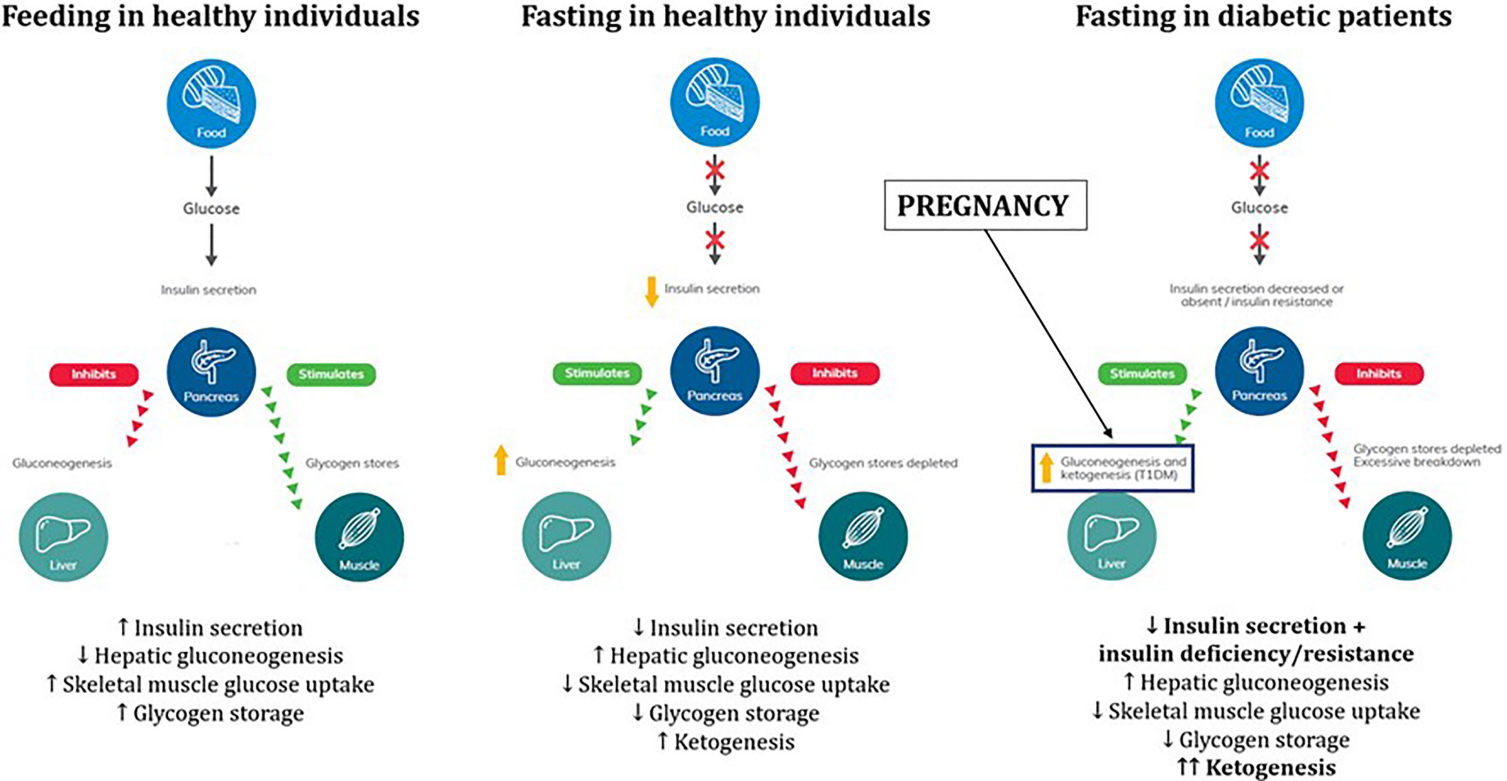
Physiology of glucose and fuel metabolism during fasting in healthy individuals, pregnancy and diabetic patients. Adapted from International Diabetes Federation (IDF) 2021 guidelines on management of diabetes during Ramadan (38). Written permission obtained by Professor Mohamed Hassanein and Professor Bachar Afandi, lead authors of the IDF 2021 guidelines.

During feeding in healthy individuals (**Figure 1**), glucose intake stimulates insulin secretion by pancreatic β-cells resulting in inhibition of hepatic gluconeogenesis (largely due to suppression of glucagon release) and stimulation of glucose uptake by hepatic, skeletal muscle and adipose tissue which can either be used as fuel or stored as glycogen or triglycerides (39).

During fasting in healthy individuals, reduced glucose intake suppresses insulin secretion and promotes glucagon release resulting in stimulation of hepatic gluconeogenesis and glycogenolysis and inhibition of skeletal muscle glucose uptake to maintain normoglycemia (40). Elevated glucagon/insulin ratio and glycogen storage depletion after prolonged fast promote lipolysis and free fatty acid β-oxidation resulting in ketogenesis as an alternative fuel source (41).

When patients with diabetes fast, reduced glucose intake suppresses insulin secretion however there is underlying pathophysiology of insulin deficiency and/or insulin resistance and thus potentially greater degree of hepatic gluconeogenesis, glycogenolysis and ketogenesis (42). This can be further aggravated during the 2^nd^ and 3^rd^ trimesters of pregnancy which is considered a period of ‘accelerated starvation’. Human placental lactogen promotes insulin resistance and inefficient glucose utilization by the mother to shift glucose delivery to the fetus, which can promote an exaggerated maternal ketogenesis response to fasting (43, 44). Hence, fasting during Ramadan in pregnant women with diabetes could represent a ‘perfect storm’ of factors culminating in potentially harmful metabolic adaptations such as ketogenesis (38).

## Ramadan Fasting and Potential Risks to the Mother

### Non-Pregnant Patients With Diabetes

There is a theoretical risk of fasting-precipitated diabetic ketoacidosis (DKA) in patients with diabetes observing Ramadan fast, however studies investigating this have been poorly designed and data conflicting although most studies suggest no increased risk of DKA. The largest study retrospectively compared DKA incidence during Ramadan with other non-Ramadan months in the UAE, including 432 episodes of DKA in 283 patients (45). There was no significant difference in incidence of DKA admissions during Ramadan and the average non-Ramadan month (3.6/month vs 3.3/month, p = 0.77). However investigators did not outline maternal fasting status. The DKAR multi-national prospective observational study (46) of 170 patients with diabetes admitted with DKA found no difference in DKA incidence or hospital length-of-stay however duration was longer during Ramadan (23.8 hours) and the month post-Ramadan (23 hours) vs pre-Ramadan (13.2 hours). All patients with DKA during Ramadan had T1DM. There was no difference in mean HbA1c between those with and without DKA. An earlier study (DKAR1) by the same group investigating 48 DKA admissions similarly showed prolonged hospital stay during Ramadan compared to the month post-Ramadan (47). In both studies, a lower incidence of DKA was found during Ramadan compared to the month post-Ramadan, with potential explanations including flow-on effect of worse glycaemic control during Ramadan or post-Ramadan festivities resulting in discursions in carbohydrate intake and glycaemic control. However, majority of participants in both studies were not fasting during Ramadan and so the association between Ramadan fasting and DKA events was not well characterized. A one-year retrospective Libyan study found lower incidence of DKA (15/month) compared to the average 19.5/month (p <0.001), with no difference in length of stay or mortality. Rates of severe DKA were non-significantly higher (26.6% vs 16.8%, p = 0.3) during Ramadan. The fasting status of patients or the local diabetic population were not indicated (48). In a study of 106 adolescents and young adults with T1DM (mean age 19 years, mean HbA1c 11.0%) of whom 92/106 (87%) completed the entire fasting month, only 1.8% developed DKA during Ramadan, comparable to institutional rates observed in non-Ramadan months (49).

Regarding impact of Ramadan fasting on glycemic control in patients with diabetes, earlier large-scale observational studies showed either no difference or reduction in HbA1c and mean BGLs (17, 50–55). Limitations included retrospective study design, reliance on patient self-reporting, and comparing pre- and post-Ramadan rather than observing changes during Ramadan. Two notable population studies were EPIDIAR (12,243 patients) (17) and CREED (3,250 patients) conducted in several Muslim-majority countries (51), which signaled concerns regarding hypoglycemia. In EPIDIAR, severe hypoglycemia (requiring hospitalization) occurred more frequently during Ramadan compared to other months (T1DM – 0.14 vs 0.03/month, p = 0.0174; T2DM – 0.03 vs 0.004/month, p <0.001). Severe hyperglycemia occurred more frequently during Ramadan in patients with T2DM (0.05 vs 0.01/month, p <0.001). As many as 42.8% of T1DM and 78.7% of T2DM patients fasted ≥15 days during Ramadan. In CREED, the average fasting duration ranged between 21-27 days and 94% of patients fasted ≥15 days during Ramadan. No significant difference was found in HbA1c pre- vs post-Ramadan. Overall hypoglycemia incidence during Ramadan was 7.1% and hypoglycemia pre-Ramadan was associated with higher odds of hypoglycemia during Ramadan (OR 7.80, 95% CI 5.31-11.45).

**Figure 2:**
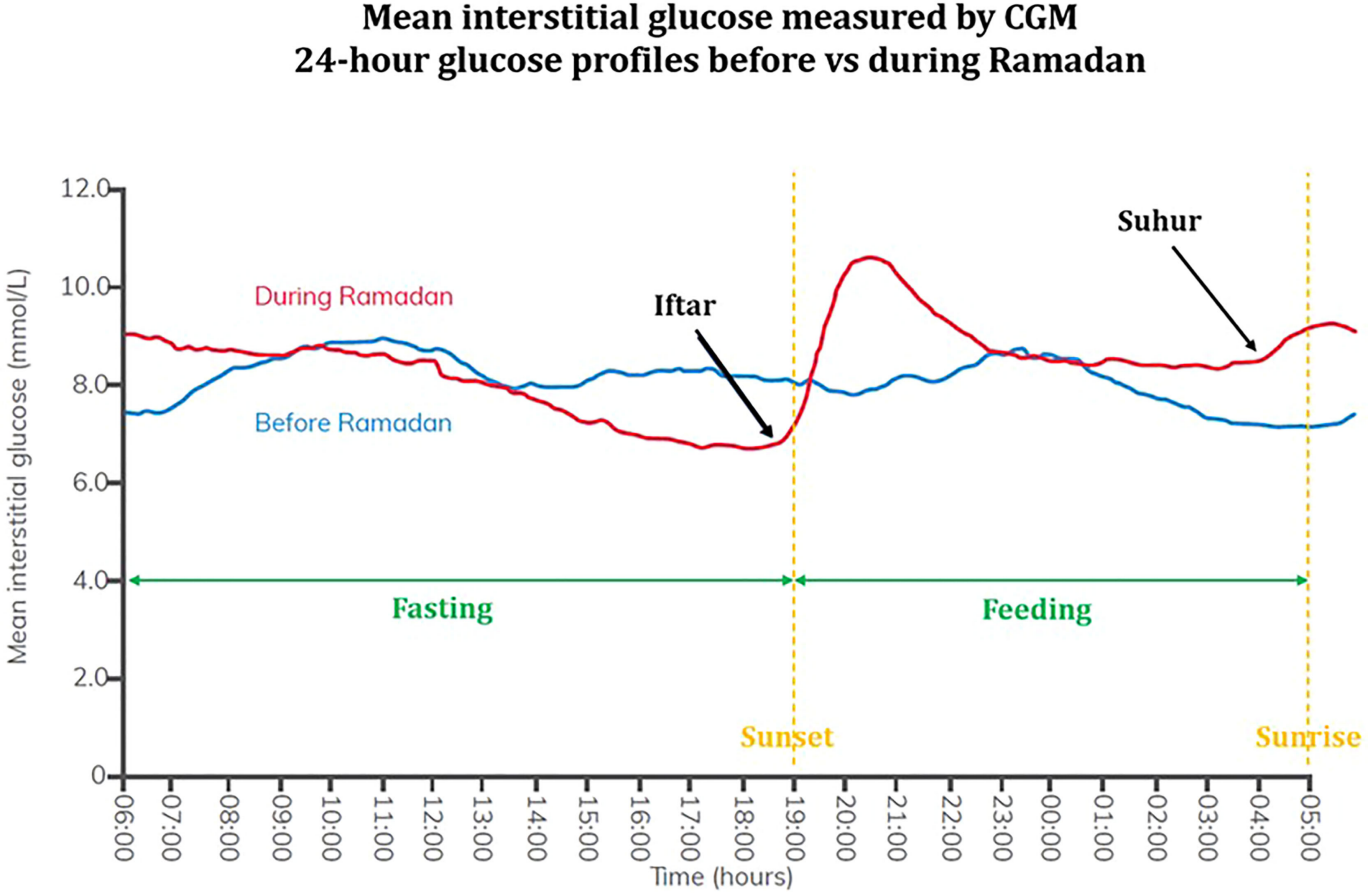
Mean interstitial 24-hour glucose profile measured by CGM in patients with type 2 diabetes before and during Ramadan. Adapted from International Diabetes Federation (IDF) 2021 guidelines on management of diabetes during Ramadan. Written permission obtained by Professor Mohamed Hassanein and Professor Bachar Afandi, lead authors of the IDF 2021 guidelines. Continuous glucose monitoring (CGM) data was obtained from patients with type 2 diabetes (n = 54) before Ramadan (blue) and during Ramadan (red). Fasting and feeding periods (green) as well as sunset and sunrise times (yellow) are indicated. Average interstitial glucose concentrations were similar between periods before and during Ramadan. Greater glycemic variability was demonstrated during Ramadan with nadir in BGLs pre-Iftar followed by peak post-Iftar, with second smaller rise post-Suhur.

More recently, small-scale prospective studies utilizing continuous glucose monitoring (CGM) have supported concerns regarding hypoglycemia (particularly fasting) and suggested greater glycemic variability in fasting patients with diabetes with BGL nadir pre-Iftar and peak post-Iftar (56–63). The largest study was conducted by Lessan et al. on 56 predominantly well-managed patients with diabetes (mean HbA1c 7.2%, 50/56 had T2DM) with minimal insulin/sulfonylurea use and 7 healthy controls (61). During Ramadan, CGM curves showed rapid rise in interstitial glucose post-Iftar, but no significant changes in mean glucose, glycemic variability and hypoglycemia frequency between patients with diabetes and controls. However, there were significantly lower BGLs pre-Iftar and higher BGLs post-Iftar when comparing fasting and non-fasting periods for patients with diabetes (**Figure 2**). One study showed no difference in hypoglycemia frequency however hypoglycemia incidence was low as patients with T1DM, insulin therapy, HbA1c >8% or history of recurrent or recent hypoglycemia were excluded (60). Two studies suggested well-controlled diabetes pre-Ramadan is associated with better glycaemic control during Ramadan (58, 61). Khalil et al. followed 21 patients with T1DM on insulin pump before and during Ramadan and found no difference in total daily insulin but redistribution with decreased basal insulin by 5-20% during daytime fast and increased prandial insulin, with no major hypoglycemic episodes (62). A randomized study of 60 patients with T1DM on sensor-augmented insulin pump +/- low-glucose suspend (LGS) found 48.6% of LGS alerts occurred in the last 3 hours of the fast and basal insulin requirements decreased by 30% (63).

### Pregnant Women With Diabetes

Data on glycemic changes in pregnant women with GDM fasting during Ramadan is scarce (**Supplementary Table 1**). The two earliest studies in Malaysia suggested Ramadan was associated with BGL-lowering. Ismail et al. retrospectively analyzed 37 pregnant women with insulin-managed diabetes during Ramadan (24 with T2DM, 13 with GDM) with mean gestational age 25 weeks (64). Median number of days fasted was 25 days. Glycemic monitoring (and thus mean BGLs, and frequency of hyper- and hypoglycaemia) was not reported however there was no self-reported hypoglycaemia during this study. Women with GDM experienced a non-significant reduction in HbA1c from 6.2% pre-Ramadan to 5.6% post-Ramadan, with no difference in patients with T2DM. Azlin et al. conducted a prospective cohort study during Ramadan on 24 pregnant women with diabetes treated with insulin, of which majority had GDM (n = 14), compared to T2DM (n = 9) and T1DM (n = 1) (65, 66). Majority of women were in their 2^nd^ trimester (54.2%) followed by 3^rd^ trimester (37.5%). Most women (79.2%) fasted ≥15 days and 33.3% were able to fast the entire month. Mean fasting BGL was significantly lower 2-weeks post-Ramadan compared to 1-week pre-Ramadan (5.34 mmol/L vs 6.16 mmol/L, p = 0.001). Mean fasting (pre-Iftar) BGLs were 5.20 mmol/L and 4.90 mmol/L during week-1 and week-4 of Ramadan. The investigators did not report hypoglycaemia frequency however and thus it is unclear whether lower BGLs during and post-Ramadan reflected better glycemic control or increased hypoglycemia.

More recent data has emerged from three small-scale prospective observational studies from the UAE utilizing blinded CGM in women with GDM fasting during Ramadan (67–69). Definitions of dysglycemia in these studies include: in-range 3.9-7.8 mmol/L, hyperglycemia 7.8-10.0 mmol/L, severe hyperglycemia >10.0 mmol/L, mild hypoglycemia 3.4 – 3.9 mmol/L, moderate hypoglycemia 2.8 – 3.3 mmol/L and severe hypoglycemia <2.8 mmol/L. There was no delineation between definitions for fasting vs post-prandial BGLs. Fasting duration was 15 hours. Afandi et al. prospectively observed 32 women with GDM, non-randomly divided into three groups: women with diet-controlled GDM pre-Ramadan (n = 10), diet-controlled GDM during Ramadan (n = 13) and diet and metformin-controlled GDM during Ramadan (n = 9) (67). Due to inter-group heterogeneity, the fairest comparison between pre-Ramadan and during Ramadan was in the diet-controlled groups. Compared to pre-Ramadan group, fasting women had lower mean BGLs (5.88 mmol/L vs 6.16 mmol/L), higher time in range (89.0% vs 78.6%), lower time in hyperglycemia (7.18% vs 19.0%), however more time in hypoglycemia (3.76% vs 2.71%) and severe hypoglycemia (0.9% vs 0%), with all Ramadan-associated hypoglycemia in the last 3 hours of the fast. Lower mean BGLs and higher time in-range during Ramadan may indicate better glycemic control during Ramadan, however this was at the cost of more frequent fasting hypoglycemia. Although mild asymptomatic hypoglycemia is not uncommon in pregnancy (70), of particular concern is the 0.9% time spent in severe hypoglycemia <2.8 mmol/L. There were no reported hospitalizations during the study however investigators did not outline whether these episodes were symptomatic or serious (i.e. requiring external assistance). Afandi et al. conducted another prospective observational study on 25 women in 2^nd^ or 3^rd^ trimester with GDM either well controlled with diet (n =14) or diet and metformin (n = 11) (68). All women were analyzed using CGM and self-monitoring blood glucose (SMBG) during Ramadan fasting. No comparisons were made between glycemic control during Ramadan and pre-Ramadan. Comparing CGM to SMBG, average BGL was 5.7 mmol/L vs 6.3 mmol/L (p <0.0001), hyperglycemia rates were 5.65% vs 14.2% (p = <0.001), hypoglycemia 4.35% vs 1.5% (p = 0.004), and severe hypoglycemia 0.9% vs 0%, with all hypoglycemia occurring in the last 3 fasting hours. This study indicated SMBG may miss a significant component of fasting hypoglycemia (including severe hypoglycemia) in Ramadan fasting women with GDM, however did not indicate frequency of clinically relevant outcomes such as hospitalizations and serious or symptomatic hypoglycemia. Hassanein et al. observed 25 women prospectively with GDM (mean gestational age 26 weeks) pre-Ramadan and during Ramadan, managed with diet alone (n = 8), metformin (n = 12), or insulin (n = 5) (69). Majority (68%) were able to fast ≥21 days. Ramadan fasting was associated with lower HbA1c (5.4% vs 5.8%, p <0.001), lower mean BGL (5.3 mmol/L vs 5.8 mmol/L, p <0.001), lower hyperglycemia frequency (13.6% vs 21.5%, p = 0.006), although more frequent hypoglycemia (9.1% vs 4.0%, p = 0.007) and no difference in time-in-range. Majority (80%) of patients developed post-Iftar hyperglycemia. Symptomatic hypoglycemia was reported in 20% of mild hypoglycemia, 86% of moderate hypoglycemia and 71% of severe hypoglycemia episodes. Serious hypoglycemia was not recorded although no hospitalizations were required for hypoglycemia.

Collectively, these three studies showed: 1) overall reduction in mean BGL and HbA1c, 2) no increase in hyperglycemia frequency (but vast majority occurring post-Iftar), and 3) increased fasting hypoglycemia frequency (almost exclusively in the last 3 fasting hours and often asymptomatic). Improved glycemic control appeared to come at the cost of increased hypoglycemia however clinical significance is unclear due to lack of reporting of symptomatic or serious hypoglycemia, although no hospitalizations were required in the 2/3 studies which reported this. These studies are all limited by small sample size, lack of randomization and generalizability of findings given studies involved highly motivated and educated women receiving extensive counselling managed in highly experienced centers in a Muslim-majority nation with considerable exposure to fasting Muslim women. No studies delineated between fasting and post-prandial glycemic control or explored the association between Ramadan fasting in women with GDM and fetal outcomes. No studies, to-date, have explored the incidence of ketosis or DKA during Ramadan fasting in pregnant women with diabetes.

## Ramadan Fasting and Potential Risks to the Fetus

Only one study published in 2021 investigated the association between maternal Ramadan exposure and neonatal outcomes in pregnant women with GDM (71). This retrospective cohort study included 345 Muslim women with singleton pregnancies between 1989 and 2010 from a single tertiary referral center in Australia. No significant effect of duration and trimester of Ramadan exposure was found on mean birthweight or macrosomia frequency. Neonatal hypoglycemia prevalence was reduced with longer Ramadan exposure (OR 0.4, p = 0.02 stratified according to 21-30 days exposure vs no exposure) after adjusting for gestational age at delivery, insulin treatment and birthweight centile. Authors suggested reduced neonatal hypoglycemia frequency without difference in birthweight may reflect better glycemic control during Ramadan however maternal glycemia was not recorded. There was a novel unexplained trend for increased neonatal hyperbilirubinemia with longer Ramadan exposure (OR 3.9, p = 0.03) and 3^rd^ trimester exposure (OR 4.3, p = 0.04). A significant limitation was lack of documentation of fasting status, i.e. Ramadan-exposed pregnancies were defined as those coinciding with Ramadan without delineating presence or duration of actual maternal fasting.

Given paucity of data on perinatal outcomes in pregnant women with diabetes fasting during Ramadan, we have summarized relevant data in healthy pregnant women. Numerous studies have explored associations between fetal outcomes and Ramadan fasting in healthy pregnant women with conflicting results. A systematic review and meta-analysis of 22 controlled studies (including 31,374 pregnancies, of which 18,920 were Ramadan-exposed) found no association with low birth weight or pre-term delivery with insufficient data to analyze association with other fetal parameters, indicating more high-quality evidence is needed to ascertain risks of maternal Ramadan fasting on the fetus (72).

Several studies investigated the association between Ramadan fasting during pregnancy and fetal outcomes (73–100). Majority of these studies are small-scale cross-sectional studies performed in resource-poor countries which exclusively included pregnancies from late 2^nd^ trimester onwards. Consistently, no association has been found with fetal artery indices using doppler ultrasonography, fetal heart rate or neonatal Apgar scores. A minority have shown associations with oligohydramnios, preterm birth, reduced placental weight, low birth weight, Cesarean section and neonatal intensive care unit admissions. Increased risk of fetal death was found in one small study in rural Africa (101) however a much larger study of 139,322 births in the Netherlands found no difference (102).

Given lack of data on fetal outcomes in pregnancies complicated by diabetes and Ramadan fasting, we have extrapolated potential mechanisms for adverse fetal effects based on existing related evidence.

As mentioned, there is elevated post-Iftar hyperglycemia in patients with diabetes during Ramadan and there is already a strong evidence basis for negative effects of maternal hyperglycemia on the fetus. These include fetal malformation in 1^st^ trimester exposed- and macrosomia, birth trauma, preterm delivery and neonatal hypoglycemia in 2^nd^ and 3^rd^ trimester exposed-pregnancies, as well as potential impacts on offspring development of obesity, T2DM and cardiovascular disease later in life (103–105). However, it is unclear whether dysglycemia for a 1-month period (e.g during Ramadan) would be sufficient to place the fetus at such risk.

As outlined, there is evidence for increased maternal fasting hypoglycemia particularly pre-Iftar but data is scarce on whether maternal hypoglycemia negatively impacts the fetus. One observational study from the United Kingdom (106) analyzed births from pregnant women with pre-existing diabetes (n = 222) matched with controls (n = 220) and found congenital anomaly/fetal death was not associated with recurrent (OR 1.1, 95% CI 0.7-1.7) or severe hypoglycemia (OR 1.3, 95% CI 0.7-2.3). A recent retrospective Turkish study compared outcomes in fetuses born to pregnant women with hypoglycemia (n = 71) and normoglycemia (n = 554) on routine screening OGTT (107). Maternal single time-point hypoglycemia was associated with reduced birth weight (2.9kg vs 3.2kg), head circumference and body length with no difference in delivery type, gestational age, preterm labour, Apgar scores and NICU admissions.

Limited existing data has not shown an association between maternal fasting ketosis and negative fetal outcomes including no association with fetal malformation risk in the Diabetes in Early Pregnancy Study (108). Although maternal DKA is a potentially catastrophic perinatal event (≥15% fetal mortality rate in small retrospective studies) (109), studies on Ramadan fasting in pregnant women or patients with diabetes have either not reported or not found increased DKA incidence.

Nutritional fetal programming (i.e. metabolic disturbances in-utero) may impact growth, development and metabolic signaling during pregnancies exposed to Ramadan fasting (110). Therefore, more studies are needed to elucidate the short- and long-term impacts of maternal Ramadan fasting on the offspring, particularly in pregnancies complicated by diabetes.

## Conclusions

In the United States and other Western countries, there is an emerging Muslim diabetic population and majority of Muslim pregnant women and patients with diabetes choose to fast during Ramadan. Hence there is an urgent need for better data, physician understanding and training to be better equipped going forward to deal with this issue.

There is a growing body of evidence according to recent small-scale CGM data in pregnant Ramadan fasting women with diabetes suggesting increased glycemic variability particularly fasting hypoglycemia however there is lack of data on important clinical outcomes such as symptomatic and serious hypoglycemia requiring hospitalization. Only one study has evaluated perinatal outcomes in pregnant women with GDM and found no effect of Ramadan exposure on mean birthweight but lower neonatal hypoglycaemia prevalence, limited by lack of documentation of maternal fasting status. More high-quality data is required regarding potential associations of Ramadan fasting with adverse maternal and fetal outcomes to better inform physician and patient decision-making, and until then, the current recommendation is against Ramadan fasting for pregnant Muslim with diabetes. The surge of literature in Muslim-majority nations is encouraging however ideally studies are performed in non-Muslim majority nations as well given differing cultural and clinician practices. Randomized-controlled trials are unlikely to have a role in this setting and thus large-scale, well-defined population-based studies should be performed including Ramadan fasting across all trimesters with clinically meaningful maternal and neonatal outcomes (e.g. symptomatic and serious hypoglycemia, hospitalizations and DKA episodes). Studies evaluating impacts of Ramadan fasting on maternal glycemic control must include BGLs during the month of Ramadan rather than comparing pre- and post-Ramadan. It is paramount that studies clearly delineate maternal fasting status, given heterogeneity in different populations regarding proportion of pregnant Muslim women and patients with diabetes who choose to fast during Ramadan. High-quality data from such studies will assist clinicians in providing evidence-based advice for mothers to help navigate a potentially challenging pregnancy.

## Data Availability Statement

Data sharing is not applicable to this manuscript as no datasets were generated in the preparation of this manuscript.

## Author Contributions

SK conceived the review, identified and appraised the relevant literature and drafted the manuscript. TD assisted in conceiving the review and critically reviewed and edited the manuscript. SK and TD both approved the final version of the manuscript. All authors contributed to the article and approved the submitted version.

## Conflict of Interest

The authors declare that the research was conducted in the absence of any commercial or financial relationships that could be construed as a potential conflict of interest.

## Publisher’s Note

All claims expressed in this article are solely those of the authors and do not necessarily represent those of their affiliated organizations, or those of the publisher, the editors and the reviewers. Any product that may be evaluated in this article, or claim that may be made by its manufacturer, is not guaranteed or endorsed by the publisher.
