# Supplementary material for: Ramadan Fasting and Maternal and Fetal Outcomes in Pregnant Women with Diabetes Mellitus: Literature Review
Source: Front Endocrinol (Lausanne). 2022 Jun 24;13:900153. doi: 10.3389/fendo.2022.900153 (PMC9263982; doi:10.3389/fendo.2022.900153)
Supplement: Supplementary file 1 [file Table_1.docx]

**TABLE 1 – Summary of studies assessing glycemic control during and pre-Ramadan fasting in pregnant Muslim women with diabetes**

| **AUTHOR** | **YEAR COUNTRY** | **STUDY DESIGN** | **STUDY POPULATION** | **BASELINE DEMOGRAPHICS** | **GLYCEMIC MONITORING** | **DIABETES TREATMENT** | **OUTCOMES** |
| --- | --- | --- | --- | --- | --- | --- | --- |
| Hassanein et al. | 2021 UAE | Prospective | GDM (n = 25) | Mean age = 31.8 years Mean gestation = 26 weeks | CGM (blinded) | Diet (n = 8) Metformin (n = 10) Metformin + Insulin  (n = 5) | **Ramadan vs Pre-Ramadan**  Mean BGL 5.3 vs 5.8 mmol/L (p <0.001)  Hypoglycemia 9.1% vs 4.0% (p = 0.007) Hyperglycemia 13.6% vs 21.5% (p = 0.006) HbA1c 5.4% vs 5.8% (p <0.001) No difference in time in-range No hospitalizations  Symptoms uncommon (20%) in mild hypoglycemia  No recording of serious hypoglycemia |
| Afandi et al. | 2019 UAE | Prospective | GDM (n = 25) | Mean age = 31 years Mean BMI = 29.3 kg/m^2^ 2^nd^ or 3^rd^ trimester only | CGM (blinded) SMBG | Diet (n = 14) Metformin (n = 11) | **CGM vs SMBG**  Mean BGL 5.7 vs 6.3 mmol/L (p <0.001 Hypoglycemia 4.4% vs 1.5% (p = 0.004) Hyperglycemia 5.6% vs 14.2% (p <0.001) No recording of hospitalizations No recording of symptomatic or serious hypoglycemia |
| Afandi et al. | 2017 UAE | Prospective | GDM (n = 32) | Mean age = 32.9 years Mean BMI = 29.5 kg/m^2^ | CGM (blinded) | Diet pre-Ramadan  (n = 10) Diet in-Ramadan  (n = 12) Metformin in-Ramadan  (n = 9) | **Ramadan vs Pre-Ramadan**  Mean BGL 6.2 vs 5.9 vs mmol/L Time in-range 89% vs 79% Hyperglycemia 7.2% vs 19% Hypoglycemia 3.8% vs 2.7% Severe hypoglycemia 0.9% vs 0% All hypoglycemia in last 3-hours of fast No hospitalizations  No recording of symptomatic or serious hypoglycemia |
| Ismail et al. | 2011 Malaysia | Retrospective | Diabetes in pregnancy managed with insulin (n = 37) T2DM (n = 24) GDM (n = 13) | Mean age = 32.1 years  Mean gestation = 25 weeks | SMBG | Short-acting insulin  (n = 6)  Short/intermediate-acting insulin  (n = 25) | **Post-Ramadan vs Pre-Ramadan**  HbA1c 5.6% vs 6.2% (non-significant) No self-reported hypoglycemic events  No glucose monitoring data recorded |
| Azlin et al. | 2011 Malaysia | Prospective | Diabetes in pregnancy managed with insulin (n = 24) GDM (n = 14)  T2DM (n = 9) T1DM (n = 1) | Majority 30-39 years old Majority 2^nd^ trimester (54%) | SMBG | Insulin (n = 24) | **Post-Ramadan vs Pre-Ramadan**  Mean fasting BGL 5.3 vs 6.2 mmol/L (p = 0.001) Hypoglycemia frequency not recorded No difference in HbA1c |

UAE = United Arab Emirates; GDM = gestational diabetes mellitus; T2DM = type 2 diabetes mellitus; T1DM = type 1 diabetes mellitus; BMI = body mass index; CGM = continuous glucose monitoring; SMBG = self-monitored blood glucose; BGL = blood glucose level; HbA1c = glycated hemoglobin.
